# Quantitative Assessment of Heteroplasmy of Mitochondrial Genome: Perspectives in Diagnostics and Methodological Pitfalls

**DOI:** 10.1155/2014/292017

**Published:** 2014-04-10

**Authors:** Igor A. Sobenin, Konstantin Y. Mitrofanov, Andrey V. Zhelankin, Margarita A. Sazonova, Anton Y. Postnov, Victor V. Revin, Yuri V. Bobryshev, Alexander N. Orekhov

**Affiliations:** ^1^Laboratory of Medical Genetics, Russian Cardiology Research and Production Complex, Moscow 121552, Russia; ^2^Laboratory of Cellular Mechanisms of Atherogenesis, Institute of General Pathology and Pathophysiology, Moscow 125315, Russia; ^3^Biological Faculty, N.P. Ogaryov Mordovian State University, Republic of Mordovia, Saransk 430005, Russia; ^4^Faculty of Medicine, School of Medical Sciences, University of New South Wales, Kensington, Sydney, NSW 2052, Australia; ^5^School of Medicine, University of Western Sydney, Campbelltown, NSW 2560, Australia; ^6^Institute for Atherosclerosis Research, Skolkovo Innovative Centre, Moscow 143025, Russia

## Abstract

The role of alterations of mitochondrial DNA (mtDNA) in the development of human pathologies is not understood well. Most of mitochondrial mutations are characterized by the phenomenon of heteroplasmy which is defined as the presence of a mixture of more than one type of an organellar genome within a cell or tissue. The level of heteroplasmy varies in wide range, and the expression of disease is dependent on the percent of alleles bearing mutations, thus allowing consumption that an upper threshold level may exist beyond which the mitochondrial function collapses. Recent findings have demonstrated that some mtDNA heteroplasmic mutations are associated with widely spread chronic diseases, including atherosclerosis and cancer. Actually, each etiological mtDNA mutation has its own heteroplasmy threshold that needs to be measured. Therefore, quantitative evaluation of a mutant allele of mitochondrial genome is an obvious methodological challenge, since it may be a keystone for diagnostics of individual genetic predisposition to the disease. This review provides a comprehensive comparison of methods applicable to the measurement of heteroplasmy level of mitochondrial mutations associated with the development of pathology, in particular, in atherosclerosis and its clinical manifestations.

## 1. Introduction

Analysis of genetic predisposition to the development of chronic diseases in humans may be a significant and innovative addition to the existing approaches to evaluation of predisposition to different pathologies, including atherosclerotic disease and cancer. Mutations of mitochondrial genome represent the novel type of genetic markers to predict the development of disease. Advisability of studies on pathogenic role of such mutations is derived from molecular mechanisms, by which they can cause defects in the protein structure of some respiratory chain enzymes and in transfer RNA (tRNA), synthesized directly in the mitochondria. This, in turn, leads to reduction in concentrations of these enzymes and tRNA or their complete dysfunction in the mitochondria. As a result, oxidative stress occurs in the cells, which is likely to contribute to the emergence and development of pathology. Typically, mutations in mitochondrial genes coding tRNA and respiratory chain proteins lead to the disruption of protein synthesis in mitochondria, which decrease the efficiency of oxidative phosphorylation and ATP production.

In contrast to the nuclear genome, mitochondrial DNA (mtDNA) is inherited strictly through the maternal line. The systems of mtDNA reparation act less efficiently than those of nuclear DNA [1]. This results in mtDNA mutation rate 10–20 times higher than nuclear DNA [2]. Mutations of mtDNA may be accumulated in certain tissue without affecting other tissue. Such a mosaic type of distribution of mitochondrial mutations may lead to focal development of pathology in organs and tissue. Most of mitochondrial mutations are characterized by the phenomenon of heteroplasmy, which is defined as the presence of a mixture of more than one type of an organellar genome within a cell or tissue. The level of heteroplasmy varies in wide range, and the expression of disease is dependent on the percent of alleles bearing mutations, thus allowing consumption that an upper threshold level may exist beyond which the mitochondrial function collapses. Typically, the emergence of mitochondrial pathology requires high levels of mutant mtDNA alleles in specific tissue exceeding 50–60% [3]. However, there is evidence that lower levels of heteroplasmy of certain mtDNA mutations that do not exceed 30–40% already significantly increase the risk of age-related chronic diseases such as left ventricular hypertrophy, atherosclerosis, coronary heart disease, and diabetes [4–6]. Therefore, both qualitative and quantitative estimations of mutant alleles in the mitochondrial genome (presence or absence of a mutation and the level of heteroplasmy, respectively) are necessary for studying the association between mitochondrial mutations and human diseases. For the quantitative determination of mutational burden of mitochondrial genome, there are a number of challenges and obstacles, which significantly limit the ability of the genetic diagnostics.

To detect and quantify mtDNA heteroplasmy levels many methodologies may be employed, including Sanger DNA sequencing [7], high performance liquid chromatography [8], pyrosequencing [5], Snapshot [9], HRM (high resolution melt profiling) [10], temporal temperature gradient gel electrophoresis (TTGE) [11], invader assay [12], amplification refractory mutation system (ARMS) [13], endonuclease method using Surveyor nuclease [14], and the next generation sequencing [15]. This review provides a comprehensive comparison of methods applicable to the measurement of heteroplasmy level of mitochondrial mutations associated with the development of pathology in humans.

## 2. Association between Heteroplasmy of Mitochondrial Mutations and Chronic Diseases

In general, there are two major pathologies, which provide the “lion's share” of morbidity and mortality in modern society and represent the key challenge to healthcare systems; these are atherosclerosis and oncopathology. The mitochondrial mutations may play mechanistic role in the development of both pathologies, and nowadays there is a growing body of evidence in support of a nonredundant role of mitochondrial factors in the pathogenesis of these diseases.

During the process of aging, mtDNA increasingly accumulates somatic mutations through multiplication of damaged DNA molecules because of limited effectiveness of the reparative mechanisms. In experiments in mitochondrial DNA polymerase-deficient mice (mtDNA mutator mice), it was demonstrated that the premature aging is the result of the increase of mtDNA mutations, which primarily alter genes encoding respiratory chain proteins, thereby reducing the functions of mitochondrial complexes I, III, and IV [16]. Thus, the failure of mitochondrial biogenesis (i.e., reduction of respiratory function) can be a major inducer of premature aging in mtDNA [17]. Due to the large number of the mitochondrial genome copies in each cell, the ratio of the mutant and wild-type mtDNA is a significant factor in a phenotype formation [18]. Moreover, the accumulation of alleles bearing mtDNA point mutations may be associated with mitochondrial dysfunction.

Nowadays, there is a limited number of studies on evaluation of mtDNA mutations in atherosclerosis. Since several early studies have demonstrated the monoclonal type of increase of vascular smooth muscle cells number in atherosclerotic lesions [19–21], a possible mechanism of the focal origin of atherosclerotic plaques may be partially explained by a local damage of cells with high level of mutated mtDNA.

In two studies, del4977mtDNA mutation (deletion of ~5 KB mtDNA region containing five tRNA genes and seven genes of respiratory chain enzymes) was analyzed, but no significant differences in the heteroplasmy levels were found between control subjects and patients with coronary atherosclerosis [22, 23]. On the other hand, the frequency of this mutation was 5-fold higher in atherosclerotic patients than in controls [23].

A mitochondrial disorder can result from the substitution, deletion, and duplication of mtDNA bases and depletion of mtDNA copies. Most of the data come from the studies on point substitute mutations. As an example, heteroplasmy level of mutation A3243G in the mitochondrial tRNA-Leu 1 gene (UUA/G) in diabetic patients with atherosclerosis was 4-fold higher compared to those age-matched nondiabetic subjects [24].

Mutation T16189C located in the hypervariable D-loop region of mtDNA has attracted attention because of its probable association with a variety of multifactorial diseases. In the study, which included 482 patients with coronary heart disease (CHD), 505 patients with type 2 diabetes, and 1,481 healthy individuals, it was shown that the prevalence of T16189C mutation was significantly higher in patients with coronary artery disease and with diabetes [25].

Recent studies have included a comprehensive analysis of mtDNA mutations in tissue of the atherosclerotic vascular wall. In two studies, 10 of 40 analyzed mutations differed in heteroplasmy level between atherosclerotic and unaffected autopsy samples of human aortic intima [5, 26]. Mutations of mtDNA associated with lipofibrous plaques were found in genes encoding 12S rRNA, tRNA-Leu, NADH-dehydrogenase subunits 1, 2, 5, and 6, and cytochrome B. From 29% to 86% of aortic intimal samples had significant differences between the atherosclerotic plaque and unaffected tissue in relation to the level of heteroplasmy for each mutation [26]. The homogenates of damaged and normal aortic intima were compared by the mean level of heteroplasmy for these 10 mutations. For five mutations (A1555G, C3256T, T3336C, G13513A, and G15059A), the mean heteroplasmy level in atherosclerotic intimal homogenates differed significantly from those in the intact tissue. It was shown that mtDNA mutation burden in intimal tissue explains at least 14% of the variability of atherosclerosis [27]. These results suggest that the mutations in human mitochondrial genome play a significant role in the development of atherosclerosis.

In the cross-sectional clinical study the association between the level of heteroplasmy for the mutation C3256T in human white blood cells and the extent of carotid atherosclerosis, as well as the presence of coronary heart disease (CHD), was investigated [28]. High-resolution B-mode ultrasonography of carotids was used to estimate the extent of carotid atherosclerosis by measuring of the carotid intima-media thickness (cIMT). Pyrosequencing was used to estimate the level of C3256T heteroplasmy. The highly significant relationship between C3256T heteroplasmy level and predisposition to atherosclerosis was revealed. In individuals with low predisposition to atherosclerosis the mean level of C3256T heteroplasmy was 16.8%, as compared to 23.8% in moderately predisposed subjects and further to 25.2% and 28.3% in significantly and highly predisposed subjects, respectively [28].

An association of mitochondrial genetic variation with the severity of carotid atherosclerosis, as assessed by carotid intima-media thickness and the presence of coronary heart disease, was analyzed [29]. Significant correlations were found between cIMT and the levels of heteroplasmy for C3256T, T3336C, G12315A, G13513A, and G15059A mutations of mtDNA. Additionally, the levels of heteroplasmy for mutations C3256T, T3336C, G12315A, G13513A, G14459A, G14846A, and G15059A correlated significantly with the size of atherosclerotic plaques visualized in any segment of carotid arteries. A regression analysis has been performed, in which the positive or negative correlation was factored for deriving the association between mtDNA mutations and atherosclerosis. The model, which included both conventional risk factors and mutations, provided 1.4-fold better explanatory level than the model based on estimation of conventional risk factors only [29].

Taken together, these findings indicate that mutations of mitochondrial genome play a substantial role in the development of atherosclerosis. However, mechanistic explanations of this role are not available yet. As an example, C3256T mutation is located in coding sequence of the MT-TL1 gene (codon recognizing UUR) which encodes tRNA leucine and is expressed at the cellular level as a reduced amount of cellular organelles and impaired protein synthesis [30–32]. Mutations G13513A, G14459A, G14846A, and G15059A occur in coding regions of genes responsible for the synthesis of respiratory chain enzymes (MT-ND5 and MT-ND6 genes encoding the subunits 5 and 6 of NADH dehydrogenase, respectively, and MT-CYB gene encoding cytochrome B). An impairment of NADH dehydrogenase activity can be expected to attenuate NADH oxidation and CoQ (ubiquinone) reduction and thus promote oxidative stress. It is obvious that the current knowledge of the mechanisms whereby mitochondrial mutations can induce and accelerate atherogenesis at the cellular and molecular level is insufficient. The further important areas of research should include the studies evaluating the mechanistic role of mtDNA mutations in cellular and molecular mechanisms of atherogenesis, the changes in levels of oxidative DNA damage by means of specific drugs as well as chemopreventive agents (i.e., antioxidants), and the effects of such interventions on the initiation and progression of atherosclerosis. This understanding is of direct clinical relevance because increased mtDNA damage can be an important pathogenic factor, an additional prognostic predictor, and a potential target of therapeutic strategies in atherosclerosis [33].

There is also the growing evidence of the role of mitochondrial mutations in oncopathology. As an example, highly significant associations were found between mtDNA mutations and the risk of stomach cancer, colon carcinoma, lung cancer, and breast cancer [34–36].

## 3. Applicable Methodologies for Quantitative Measurement of mtDNA Heteroplasmy Level

The above considerations raise an important methodological problem, since quantitative evaluation of a mutant allele of mitochondrial genome may be a keystone for diagnostics of individual genetic predisposition to the disease, including atherosclerosis and oncopathology. Routine methods of direct sequencing, like Sanger sequencing, are generally inappropriate due to erroneous results in the case of polynucleotide sequences, very often observed in mtDNA, although such method has been used earlier for the measurement of heteroplasmy level [7]. As a rule, sequence analysis of heteroplasmy around 50% provides clear results in terms of the presence of heteroplasmy, but not in a quantitative manner. Lower level of mtDNA heteroplasmy is often undetectable by direct sequencing. As an example, Meierhofer et al. have used denaturing high performance liquid chromatography to rapidly screen the entire mtDNA for mutations; this approach yielded straightforward interpretation of results with a detection limit down to 1% mtDNA heteroplasmy. However, direct sequencing analysis has become informative only after collection and reamplification of low degree heteroduplex peak fractions [8].

At present, all existing approaches to the detection of mtDNA allelic variants can be divided into several groups:

(1) enzymatic approaches:
  - restriction fragment length polymorphism (RFLP);
  - amplified fragment length polymorphism (AFLP);
  - cleavase fragment length polymorphism (CFLP);
  - enzymatic mutation detection using the bacteriophage resolvase (EMD);
  - analysis based on the ligase reaction (LDR, LCR, padlock);
  - invasive oligonucleotide cleavage (Invader);
  - random amplification of polymorphic DNA (RAPD, AP-PCR);
  - PCR with direct termination (DT-PCR);
  - allele-specific PCR (AS-PCR);
(2) chemical methods:
  (i) chemical cleavage of heteroduplexes;
  (ii) chemical ligation;
(3) methods based on the different electrophoretic mobility of DNA polymorphisms:
  (i) analysis of the conformation of single-stranded fragments (SSCP);
  (ii) heteroduplex analysis;
  (iii) DNA sequencing;
(4) detection of the solid phase:
  (i) hybridization to oligonucleotide arrays;
  (ii) fiberoptic DNA hybridization analysis;
  (iii) elongation of immobilized primers (minisequencing);
  (iv) pyrosequencing;
(5) chromatographic methods:
  (i) denaturing HPLC;
(6) physical methods:
  (i) mass spectrometry;
  (ii) resonance fluorescence quenching using fluorescence resonance energy transfer (FRET).

Subdivision of these methods into above groups is rather nominal, primarily because in most cases a combination of different methods is used (e.g., all of the physical methods also use enzymatic reactions).

The choice of the optimal approach depends on the specific objective of the study. Historically, widely applied methods for the detection of DNA polymorphisms employed restriction enzymes and were limited to detection of changes in the restriction sites. Later, other enzymatic approaches have been developed. Although they are currently implemented mainly in a high-performance design, a good portion of data indicates the possibility of their expansion and automation. The advantages of these methods are the relatively low cost of the analysis, which is independent of the number of samples, and relative easiness of implementation. However, such methods are time-consuming; that should be regarded as obvious flaw.

Each of the above-mentioned strategies has its own advantages and disadvantages. For example, HRM, HPLC, and endonuclease method may be excellent for qualitative detection of heteroplasmy itself, but they have insufficient resolution for the quantitative measurement of mtDNA heteroplasmy level. Pyrosequencing and next generation sequencing provide high accuracy for the quantitative measurements of heteroplasmy levels but are extremely expensive, thus limiting the possibility of routine analysis of a large number of samples.

Table 1 provides an overview of the most informative methods used to quantify heteroplasmy level of mtDNA mutations. Among the quantitation methods based on the PCR, Snapshot minisequencing has insufficient resolution in measuring the content of the mutant allele of less than 5% [37]. ARMS strategy (amplification refractory mutation system) also implies the use of fluorescent dye for the quantitative determination of the mutant allele by real-time PCR. Experiments using allele-specific PCR and real-time detection of heteroplasmy level by incorporating of CYBR Green intercalating fluorescent dye have demonstrated high reproducibility of measurement (with correlation coefficient of 0.994) for the analysis of picogram amounts of total DNA obtained from different sources and the possibility of quantifying mtDNA heteroplasmy levels up to 0.5% [38]. Furthermore, ARMS strategies, unlike pyrosequencing and next generation sequencing, are not very expensive, which makes them ideal for the analysis of small samples (Table 1).

Currently, the most versatile methodology for the quantitative measurement of mtDNA heteroplasmy level is qPCR employing fluorescence detection. To detect the presence of specific nucleotide sequences using fluorescence detection in real time, there are several techniques, the most known of which are the ones with intercalating dye SYBR Green I and methods with specific degradable oligonucleotide probes (TaqMan) and hairpin structures (Scorpions) (Table 2).

Summarizing the advantages and disadvantages of this approach, it should be noted that the use of DNA probes in some specific form is the most preferable to increase the specificity of analysis of mtDNA heteroplasmy. However, the disadvantages of the above described approach relate to a high cost of probes, and accordingly, the primers and amplification process are expensive as well. At the same time, the use of intercalating agents is very simple and cheap. The need for selection of specific primers and probes seems to be a limitation factor, but now the number of commercially available primers steadily increases, and their efficiency has already been validated. Thus, the use of intercalating agents becomes highly attractive.

However, the use of intercalating dye SYBR Green I and hairpin structures (Scorpions) has certain limitations that prevent the wide use for quantitative measurement of mtDNA heteroplasmy, such as

- low specificity and frequent false-positive results for the intercalating dye SYBR Green I, the inconvenience of job associated with the need to carry out the test in two separate tubes;
- low efficiency of reaction and, consequently, low sensitivity of hairpin structures method (Scorpions).

A widely recognized method of mutations detection is the use of the energy transfer through the fluorescent resonance phenomenon (FRET), which is used, for example, in analysis systems based on TaqMan-type and similar probes [39]. TaqMan analysis is based on the 5′-nuclease activity of Taq-polymerase, which cleaves nucleotides of the probes hybridized to DNA oligonucleotide. Two TaqMan probes are required; one probe should be complementary to the wild allele and the other one to the polymorphic variant. These probes have different fluorescent dyes at the 5′-end and fluorescence quenchers at the 3′-end. When the probes are inactive, the quenchers interact with the dye by FRET mechanism, inhibiting fluorescence activity. At the primers annealing step during qPCR, TaqMan probes hybridize with the DNA molecule. In the untwisting step, 5′-end of the dye is cleaved by Taq-polymerase 5′-nuclease activity, which leads to an increase in fluorescence of the dye. Base mismatches in the probes lead to the cleavage of a probe without any dye release. The genotype of the sample is determined by the intensities of the emission of two different dyes ratio, thus allowing to calculate the proportion of mutant allele in heteroplasmic sample.

Considering the presence of two alleles (normal and mutant) within the mitochondrial genome in the same sample in an unknown proportion, the most appropriate approach for the development of RT-PCR method of mtDNA heteroplasmy quantitative measurement is the use of TaqMan probes. As for SYBR Green method, it can be used for the cases when the multiplex measurement of heteroplasmy level of some mutations would be impossible.

## 4. Conclusion

At present, it is clear that mitochondrial factor may play a pivotal role in susceptibility, induction, and progression of chronic diseases, including atherosclerosis and oncopathology. However, the impact of certain mtDNA mutations to the development of pathology is widely unknown. More studies focused on the assessment of new heteroplasmic mtDNA mutations affecting mitochondrial function in relation to atherosclerosis and oncopathology are definitely required. The deep knowledge about the pathophysiological relevance of each disease-associated mtDNA mutations is of great importance.

Among the various modern methods for quantitative assessment of mtDNA heteroplasmy level, the qPCR method based on TaqMan technologies seems to be the most promising for the purposes of clinical diagnostics. The simplicity of sequences design, low price, wide availability of equipment, and the simplicity of the analysis itself can make it available for clinical practice. In parallel, an informative pyrosequencing method may be further considered as the “gold standard” for the measurements of mtDNA heteroplasmy for gaining reference values.

## Figures and Tables

**Table 1:** Comparison of methods for the quantitative analysis of mtDNA heteroplasmy.

Method	Advantages	Disadvantages
Invasive cleavage of oligonucleotide probe (invader assay)	(1) Allows SNP-analysis in a single reaction tube (2) Allows the use of automatic setup of the reaction	(1) Requires a long incubation time (3-4 h) (2) Big amount of genomic DNA (20–100 ng) in a single reaction (3) The laborious selection of allele-specific probes

Pyrosequencing	(1) Allows to estimate the exact nucleotide sequence and its changes (2) The possibility of automation (3) Quick performance (4) Can be used for SNP-analysis (5) The ability to analyze complex secondary structures	(1) Requires special equipment (2) Limited read length (3) The quality and effectiveness of four enzymes used in reactions are critical for the accuracy of measurements (4) Enzymes can lose activity (5) Sample dilution may reduce the read length

Real-time amplification refractory mutation system for quantitative PCR analysis	(1) Simple (2) Single-stage (3) Fast (4) Nonradioactive (5) Sensitive (6) Specific	(1) It is difficult to find the optimal allele-specific primers (2) It is difficult to find the optimal mode for RT PCR to ensure proper hybridization of allele-specific primers

454 sequencing (Roche)	(1) High read length (2) Relatively low cost of the instrument and reagents for the single run of GS junior titanium	(1) High cost of the instrument and reagents for the single run of GS FLX titanium (2) High cost per 1 Mb read (3) High consumption of fluids passed through the flow cell

Illumina sequencing with Illumina MiSeq	(1) Relatively low cost of the instrument and reagents for the single run (2) The lowest cost per 1 Mb read among small platforms (3) The fastest Illumina run time	(1) Relatively few reads (2) Higher cost per 1 Mb read as compared to other Illumina platforms

Illumina HiScanSQ	(1) Allows versatile genomic research and is scalable in future	(1) Higher cost per 1 Mb read than HiSeq for large amounts of data

Illumina GAIIx	(1) Lower instrument cost than HiSeq (2) The large number of publications using this instrument	(1) Higher cost per 1 Mb read than HiSeq

Illumina HiSeq 1000 and 2000	(1) The largest number of reads (2) Maximum sequencing output per 1 day and 1 run (3) The lowest price for 1 Gb read among all NGS platforms	(1) High instrument cost (2) High computation needs

Applied Biosystems SOLiD sequencing	(1) Each lane of Flow-Chip can be run independently (2) The highest sequencing accuracy (3) The ability to rescue failed sequencing cycles (4) 96 validated barcodes per lane (5) Throughput of 10–15 Gb/day for SOLiD-5500 and 20–30 Gb/day for SOLiD 5500XL	(1) High-performance devices have become available only from 2011 (2) Relatively short reads (3) Increased number of gaps in the assembly of genomes (4) High instrument cost for SOLiD-5500XL

HRM analysis	(1) Low consumption of reagents with minor losses: it takes 20 *μ*L of PCR mix for the analysis of each sample, eliminating the need for HPLC-solvents or DGGE-gels (2) The stage of highly sensitive analysis of the melting curves can be added in the end of the PCR for immediate analysis (3) Unlike DHPLC, it does not require thermal optimization (4) Low consumption of samples: after HRM-analysis PCR products can be used for Sanger sequencing (5) High resolution for accurate and reproducible results	(1) Method is not adapted for the quantitative analysis of mitochondrial mutations

TGGE	(1) Does not require denaturing agents (2) High reproducibility (3) The ability to analyze fragments with a size of up to 1000 bp	The exact nature of the nucleotide changes is unknown

HPLC	(1) High accuracy (2) The ability to analyze very small amounts of DNA (3) High separation efficiency (4) Flexibility to alter the conditions of separation (5) Rapid analysis (6) Comparative simplicity of instrumentation (7) Automation ability	(1) High cost of columns (2) The exact nature of changes in nucleotide sequence remains unknown (3) Sequencing is required to determine the nature of the mutation (4) Difficult identification of heterozygotes

Endonuclease method using Surveyor nuclease	(1) Simplicity (2) Special kits for mutations detection	(1) Qualitative estimation

Sanger sequencing	(1) Using dideoxynucleotides with fluorescent labels with different emission wavelengths allows to carry out the reaction in a single tube (2) The ability to analyze 500–1000 bp sequence in a single run	(1) Complexity of the electrophoretic separation of fragments (2) Low throughput compared with NGS methods (3) The loss of accuracy when reading large fragments

Snapshot	(1) A reliable method to identify uncharacterized nucleotide damage (2) Allows to subsequently determine the untimely stop codons, missense, or silent mutations (3) Can be used for SNP analysis (4) Automation ability	(1) Relativeness of the results (2) Low accuracy of the data (3) Requires valuation of results

**Table 2:** The basic methods suitable for quantitative measurement of mtDNA heteroplasmy based on qPCR with fluorescence detection.

	Principle of operation	Advantages	Disadvantages
Nonspecific methods			
Intercalating dyes (SYBR Green I and others)	The fluorescence signal increases due to an intercalating dye binding to double-stranded DNA	High level of signal, the same approach to the design of various experiments	False positive results are possible due to primer-dimers or nonspecific amplicons, so these methods are not immediately suitable for diagnostic purposes
Amplifluor	Allele-specific PCR reveals a hairpin structure of fluorescently labeled oligonucleotides		
Lux	The fluorescence signal changes when the fluorescently labeled primer is included in the PCR product		
DzyNA-PCR	Binding of the probe with the PCR products generates restriction enzyme recognition site		
Specific methods			
TaqMan	The dye and the quencher are separated by 5′-exonuclease activity of Taq-polymerase	High signal level, simplicity of design, and synthesis	Problems with the discrimination of some sequences
Molecular beacons	Dye and quencher are separated after binding of the probe to the amplicon and disclosure of the hairpin	Good signal-to-noise ratio	The low level signal due to reaction kinetics
Eclipse	Similar to TaqMan, but is resistant to exonuclease activity of the polymerase; a signal is generated after binding of the probe to the amplicon	High signal level, simplicity of design, and synthesis	Poor signal-to-noise ratio, problems with the discrimination of some sequences
Hyb probes	Two dyes are converging after binding of the probes to the amplicon, the signal is generating by the FRET mechanism	Undefined	Poor signal-to-noise ratio, not suitable for all devices, the reaction requires a trimolecular interaction
Scorpions	The probe-primer is included in the PCR product, the signal is generated after disclosure of the hairpin and binding of the free end of the probe to the amplicon	Good signal-to-noise ratio, monomolecular reaction	Relatively expensive synthesis
